# Dataset of "true mangroves" plant species traits

**DOI:** 10.3897/BDJ.5.e22089

**Published:** 2017-12-29

**Authors:** Aline Ferreira Quadros, Martin Zimmer

**Affiliations:** 1 Leibniz Centre for Tropical Marine Research, Bremen, Germany

**Keywords:** Mangroves, Rhizophoraceae, leaf traits, plant traits, halophytes

## Abstract

**Background:**

Plant traits have been used extensively in ecology. They can be used as proxies for resource-acquisition strategies and facilitate the understanding of community structure and ecosystem functioning. However, many reviews and comparative analysis of plant traits do not include mangroves plants, possibly due to the lack of quantitative information available in a centralised form.

**New information:**

Here a dataset is presented with 2364 records of traits of "true mangroves" species, gathered from 88 references (published articles, books, theses and dissertations). The dataset contains information on 107 quantitative traits and 18 qualitative traits for 55 species of "true mangroves" (*sensu* Tomlinson 2016). Most traits refer to components of living trees (mainly leaves), but litter traits were also included.

## Introduction

The vegetation of mangrove forests is loosely classified as "true mangroves" or "mangrove associates". True mangroves are woody plants, facultative or obligate halophytes (Wang et al. 2011). "True mangroves" are defined by Tomlinson (2016) as plant species that 1) occur only in mangrove forests and are not found in terrestrial communities; 2) play a major role in the structure of the mangrove community, sometimes forming pure stands; 3) have morphological specialisations to the mangrove environment; 4) have some mechanism for salt exclusion. Other notable specialisations of mangrove plants include: aerial roots to counteract the anaerobic sediments, support structures such as buttresses and above-ground roots, low water potentials and high intracellular salt concentrations, salt-excretion through leaves and buoyant, viviparous propagules (Duke et al. 1998).

Following Tomlinson (2016), all species of genera *Avicennia*, *Lumnitzera*, *Bruguiera*, *Ceriops*, *Kandelia*, *Rhizophora* and *Sonneratia*, plus the species *Nypa fruticans* and *Laguncularia racemosa*, are considered as "true mangroves" and are the major components of mangrove forests worldwide. Other species, such as *Acrostichum aureum*, *Aegiceras corniculatum*, *Osbornia octodonta* et al., are also "true mangroves" but considered as minor components of mangrove forests (Tomlinson 2016).

Mangrove forests are highly threatened worldwide (Duke et al. 2007) and conservation efforts face the lack of a good understanding of mangrove community structure and ecosystem processes. With this gap in mind, literature on mangrove trees was reviewed and a dataset of traits was assembled, with the aim of contributing to future studies of mangroves using a functional trait perspective and also to allow the inclusion of mangrove trees in future comparative studies of plant ecology and resource-acquisition strategies.

## Geographic coverage

### Description

Global

## Taxonomic coverage

### Description

This dataset contains traits for 55 species of "true mangroves". To standardise the spelling of species' names, The Plant List (2013) was followed. Some species listed below are currently considered as synonyms in The Plant List (e.g. *Avicennia alba* is currently a synonym of *Avicennia marina*). However, they were chosen to be included under the names given by the authors to allow the tracking of the original information. All records of Ceriops tagal var. australis were included as *Ceriops australis*, and Ceriops tagal var. tagal was included as *Ceriops tagal* following Ballment et al. (1988).

### Taxa included

**Table:** 

Rank	Scientific Name	
species	*Acanthus ilicifolius*	
species	*Acrostichum aureum*	
species	*Aegialitis annulata*	
species	*Aegialitis rotundifolia*	
species	*Aegiceras corniculatum*	
species	*Avicennia alba*	
species	*Avicennia bicolor*	
species	*Avicennia eucalyptifolia*	
species	*Avicennia germinans*	
species	*Avicennia integra*	
species	*Avicennia lanata*	
species	*Avicennia marina*	
species	*Avicennia officinalis*	
species	*Avicennia rumphiana*	
species	*Avicennia schaueriana*	
species	*Bruguiera cylindrica*	
species	*Bruguiera exaristata*	
species	*Bruguiera gymnorhiza*	
species	*Bruguiera hainesii*	
species	*Bruguiera parviflora*	
species	*Bruguiera rhynchopetala*	
species	*Bruguiera sexangula*	
species	*Camptostemon schultzii*	
species	*Ceriops australis*	
species	*Ceriops decandra*	
species	*Ceriops tagal*	
species	*Excoecaria agallocha*	
species	*Kandelia candel*	
species	*Kandelia obovata*	
species	*Laguncularia racemosa*	
species	*Lumnitzera littorea*	
species	*Lumnitzera racemosa*	
species	*Nypa fruticans*	
species	*Osbornia octodonta*	
species	*Pelliciera rhizophorae*	
species	*Rhizophora apiculata*	
species	*Rhizophora harrisonii*	
species	*Rhizophora lamarckii*	
species	*Rhizophora mangle*	
species	*Rhizophora mucronata*	
species	*Rhizophora racemosa*	
species	*Rhizophora samoensis*	
species	*Rhizophora stylosa*	
species	*Scyphiphora hydrophylacea*	
species	*Sonneratia alba*	
species	*Sonneratia apetala*	
species	*Sonneratia caseolaris*	
species	*Sonneratia griffithii*	
species	*Sonneratia gulngai*	
species	*Sonneratia hainanensis*	
species	*Sonneratia lanceolata*	
species	*Sonneratia ovata*	
species	*Xylocarpus granatum*	
species	*Xylocarpus mekongensis*	
species	*Xylocarpus moluccensis*	

## Traits coverage

**Figure 1.**
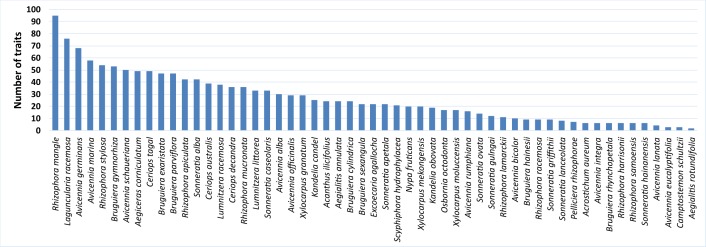
Number of traits available per mangrove species.

This dataset contains 18 qualitative traits (Table 1) and 107 quantitative traits (Table 2). The number of records per species and trait is shown in Suppl. material 1. The number of traits available per species varies from 2 to 95 and is shown in Fig. 1.

**Remarks on data collection**:

When data was provided for young leaves and mature leaves, only mature leaves were used. When studies reported traits from the same species from different locations, all locations were considered as separate records in the database. Studies that reported a range of maximum and minimum values were also added as separate records. Leaves collected from the ground were not used for measurement of traits. For leaf litter traits, data were used where authors reported using "senescent leaves", or "yellow leaves" that could be easily detached from the trees.

To facilitate the comparison of mangrove traits with those from other studies and datasets, the same trait names were used as in the TRY Database of plant traits (KATTGE et al. 2011) whenever possible.

## Usage rights

### Use license

Open Data Commons Attribution License

## Data resources

### Data package title

Mangrove plants traits

### Resource link

https://zenodo.org/record/802990

### Alternative identifiers

DOI: 10.5281/zenodo.802990

### Number of data sets

1

### Data set 1.

#### Data set name

Mangrove plants trait dataset

#### Data format

CSV file

#### Number of columns

10

#### Download URL

https://zenodo.org/record/802990

#### 

**Data set 1.** 

Column label	Column description
Compartment	Categorical. Describes whether the trait refers to the living plant (TREE), or to the litter (LITTER).
Organ	Categorical. Indicates to which plant organ the trait refers (LEAF, ROOT, BARK, FLOWER, DISPERSAL UNIT, SEED) or if it refers to the whole plant (TREE).
Trait name	Trait name
Trait value	Trait value as given in the publication
Remarks	Any important remark about that particular value
Plant species name	Species name as given in the publication
Trait type	Categorical. Describes whether the trait is QUANTITATIVE or QUALITATIVE
Trait unit	Specifies the unit of quantitative traits (e.g. percentage, mg per g, mm, g)
Source	Reference for the trait value
Record number	Sequential record number

## Supplementary Material

Supplementary material 1 Matrix of traits per species showing the number of records per each combination. Data type: phylogenetic
File: oo_176975.xlsx Aline Ferreira Quadros, Martin Zimmer

## Figures and Tables

**Table 1.** Detailed list of qualitative traits and respective references.

**Trait name**	**Type of information**	**Possible values**	**References**
dispersal unit floating capacity in freshwater	categorical	floater; sinker	Clarke et al. 2001
dispersal unit floating capacity in saltwater	categorical	floater; sinker	Clarke et al. 2001, Giesen et al. 2007
dispersal unit orientation in water	categorical	prone; prone to vertical; vertical	Clarke et al. 2001
dispersal unit shape	categorical	tear-drop; ovoid, round; long curved; long; ellipsoidal; obovate; flattened-round	Giesen et al. 2007
dispersal unit size class	ordinal	I = < 0.1 cm^3^; II = < 1 cm^3^; III = < 10 cm^3^; IV = <100 cm^3^; V = < 1000 cm^3^	Duke et al. 1998
germination type	categorical	epigeal; hypogeal	Clarke et al. 2001, Soepadmo et al. 2002, Tomlinson 1986
leaf emergences (pubescence)	binary	yes; no	Giesen et al. 2007, NParks 2017, Reef and Lovelock 2015, Sheue et al. 2003
plant growth form	categorical	shrub/small tree; tree	Giesen et al. 2007
plant position in the intertidal	ordinal	L = low; M = mid; H = high; ML = middle to low; HM = high to middle; HML = high, middle and low	Clough 1992, Duke et al. 1998
plant preferred substrate	categorical	Sand; clay; mud; riverbanks; mud/sand/peaty soils; mudflat/sand/calcareous; sand/mud; soft fine-grained;	Giesen et al. 2007
plant tolerance to drought	ordinal	1 = very low; 2 = low; 3 = mid; 4 = high; 5 = very high;	Clough 1992
plant tolerance to low temperature	ordinal	1 = very low; 2 = low; 3 = mid; 4 = high; 5 = very high;	Clough 1992
plant tolerance to salt	ordinal	1 = very low; 2 = low; 3 = mid; 4 = high; 5 = very high; or low; mid; high	Clough 1992, Reef and Lovelock 2015
plant tolerance to shade	binary	tolerant; intolerant	Smith 1992
presence of salt glands	binary	yes; no	NParks 2017, Reef and Lovelock 2015, Sheue et al. 2003
root type	categorical	non_aerial; pneumatophore; buttresses_knees; buttresses; knees; prop	Duke et al. 1998, Tomlinson 2016
sexual type	categorical	hermaphrodite; androdioecious; monoecious	Tomlinson 1986
type of embryo development	categorical	cryptoviviparous; viviparous; recalcitrant; non-viviparous	Clarke et al. 2001, Sahu et al. 2016, Farnsworth 2000, Tomlinson 1986

**Table 2.** List of quantitative traits available in the dataset and respective references of trait values.

bark carbon (C) content per unit bark dry mass	Koch 2002
bark carbon/nitrogen (C/N) ratio	Koch 2002
bark litter nitrogen (N) content per unit bark dry mass	Nordhaus 2004
bark litter carbon (C) content per unit bark dry mass	Nordhaus 2004
bark litter carbon/nitrogen (C/N) ratio	Nordhaus 2004
bark nitrogen (N) content per unit bark dry mass	Koch 2002
dispersal unit length	Clarke et al. 2001, Duke and Jackes 1987, Giesen et al. 2007, Hogarth 1999, NParks 2017, Oliveira 2005, Soepadmo et al. 2002, Van der Stocken et al. 2015, Tomlinson 2016
dispersal unit litter C/N ratio	Nordhaus 2004
dispersal unit litter carbon (C) content per unit dry mass	Nordhaus 2004
dispersal unit litter nitrogen (N) content per unit dry mass	Nordhaus 2004, Reise 2003
dispersal unit litter phosphorus (P) content per unit dry mass	Reise 2003
dispersal unit litter potassium (K) content per unit dry mass	Reise 2003
dispersal unit litter sodium (Na) content per unit dry mass	Reise 2003
dispersal unit width	Soepadmo et al. 2002
flower litter carbon (C) content per flower dry mass	Nordhaus 2004
flower litter CN ratio	Nordhaus 2004
flower litter nitrogen (N) content per flower dry mass	Nordhaus 2004
leaf acid detergent fib content per unit dry mass	Amiri 2014
leaf area	Arrivabene et al. 2014, Ball 1988, Lin and Wang 2001, Medina and Francisco 1997, Saenger and West 2016, Yuanyue et al. 2009, Medina et al. 2001, Okello et al. 2014, Reise 2003
leaf area per leaf mass (SLA)	Choong et al. 1992, Medina and Francisco 1997, Medina et al. 2001, Arrivabene et al. 2014, Ball 1988, Lin and Wang 2001, Medina and Francisco 1997, Saenger and West 2016, [Bibr B3680914][Bibr B3680842]
leaf ash content per leaf dry mass	Lacerda et al. 1986
leaf boron (B) content per leaf dry mass	Christofoletti et al. 2013
leaf calcium (Ca) content per leaf area	Wang et al. 2011
leaf calcium (Ca) content per leaf dry mass	Ahmed et al. 2010, Bernini et al. 2006, Christofoletti et al. 2013, Feller 1995, Medina et al. 2001, Woodroffe et al. 1988
leaf carbon (C) content per leaf dry mass	Koch 2002, Feller 1995, Medina et al. 2001, Nordhaus et al. 2011
leaf carbon/nitrogen (C/N) ratio	Ahmed et al. 2010, Chen and Ye 2008, Koch 2002, Medina et al. 2001, Nordhaus et al. 2011, Rao et al. 1994, Schmitt 2006
leaf cellulose content per leaf dry mass	Christofoletti et al. 2013
leaf chlorine (Cl) content per leaf dry mass	Lacerda et al. 1986, Tong et al. 2006
leaf copper (Cu) content per leaf dry mass	Bernini et al. 2006, Feller 1995, Christofoletti et al. 2013
leaf crude fiber content per leaf dry mass	Amiri 2014,Chen and Ye 2008, Choong et al. 1992, Lacerda et al. 1986, Tong et al. 2006
leaf cuticula thickness	Arrivabene et al. 2014, Das and Ghose 1996
leaf dry mass	Arrivabene et al. 2014, Medina et al. 2001, Saenger and West 2016, Lin and Wang 2001, Zimmer (unpublished data)
leaf dry mass per area (LMA)	Arrivabene et al. 2014, Ball 1988, Johnstone 1981, Lin and Wang 2001, Medeiros and Sampaio 2013, Medina et al. 2001
leaf energy content per leaf dry mass	Saenger and West 2016
leaf hemi-cellulose content per leaf dry mass	Christofoletti et al. 2013
leaf intercellular CO2 concentration	Mehlig 2001
leaf iron (Fe) content per leaf dry mass	Bernini et al. 2006, Feller 1995, Medina et al. 2001, Christofoletti et al. 2013
leaf length	Duke and Jackes 1987, Giesen et al. 2007, Soepadmo et al. 2002
leaf length/width ratio	Medina et al. 2001
leaf lifespan	Burrows 2003, Duke et al. 1984, Ellison 2002, Ellison and Farnsworth 1996, Gill and Tomlinson 1971, Khan et al. 2009, Lee 1991, Medeiros and Sampaio 2013, Mehlig 2001, Moryia et al. 1988, Saenger and West 2016, Sharma et al. 2012, Steinke 1988, Steinke and Rajh 1995, Tong et al. 2006, Wang and Lin 1999, Wang’ondu et al. 2013, Wium-Andersen 1981, Wium-Andersen and Christensen 1978
leaf lignin content per leaf dry mass	Christofoletti et al. 2013
leaf litter boron (B) content per leaf dry mass	Christofoletti et al. 2013
leaf litter calcium (Ca) content per leaf dry mass	Christofoletti et al. 2013, Woodroffe et al. 1988
leaf litter carbon (C) content per leaf dry mass	Herbon and Nordhaus 2013, Nordhaus 2004, Nordhaus et al. 2011
leaf litter carbon/nitrogen (C/N) ratio	Herbon 2011, Herbon and Nordhaus 2013, Micheli 1993, Nordhaus 2004, Nordhaus et al. 2011, Rao et al. 1994
leaf litter cellulose content per leaf dry mass	Christofoletti et al. 2013
leaf litter copper (Cu) content per leaf dry mass	Christofoletti et al. 2013
leaf litter energy content per leaf dry mass	Nordhaus 2004
leaf litter hemi-cellulose content per leaf dry mass	Christofoletti et al. 2013
leaf litter iron (Fe) content per leaf dry mass	Christofoletti et al. 2013
leaf litter lignin content per leaf dry mass	Christofoletti et al. 2013
leaf litter lignin/N ratio	Gleason and Ewel 2002
leaf litter magnesium (Mg) content per leaf dry mass	Christofoletti et al. 2013, Woodroffe et al. 1988
leaf litter manganese (Mn) content per leaf dry mass	Christofoletti et al. 2013
leaf litter nitrogen (N) content per leaf dry mass	Christofoletti et al. 2013, Herbon and Nordhaus 2013, Nordhaus 2004, Nordhaus et al. 2011, Reise 2003, Steinke et al. 1993, Woodroffe et al. 1988
leaf litter organic matter content per leaf dry mass	Micheli 1993
leaf litter phenolics content (polyphenol) per leaf dry mass	Christofoletti et al. 2013
leaf litter phosphorus (P) content per leaf dry mass	Christofoletti et al. 2013, Reise 2003, Steinke et al. 1993, Woodroffe et al. 1988
leaf litter potassium (K) content per leaf dry mass	Christofoletti et al. 2013, Reise 2003, Steinke et al. 1993, Woodroffe et al. 1988
leaf litter sodium (Na) content per leaf dry mass	Reise 2003, Woodroffe et al. 1988
leaf litter sulphur (S) content per leaf dry mass	Christofoletti et al. 2013
leaf litter tannins content per leaf dry mass	Micheli 1993, Steinke et al. 1993
leaf litter toughness	Micheli 1993
leaf litter water content per leaf dry mass	Micheli 1993
leaf litter zinc (Zn) content per leaf dry mass	Christofoletti et al. 2013
leaf magnesium (Mg) content per leaf dry mass	Bernini et al. 2006, Christofoletti et al. 2013, Feller 1995, Medina et al. 2001, Woodroffe et al. 1988
leaf manganese (Mn) content per leaf dry mass	Bernini et al. 2006, Feller 1995, Medina et al. 2001, Christofoletti et al. 2013
leaf maximum water use efficiency	Mehlig 2001
leaf nitrate (NO3-) content per leaf dry mass	Koch 2002
leaf nitrogen (N) content per leaf area	Wang et al. 2011
leaf nitrogen (N) content per leaf dry mass	Ahmed et al. 2010, Amiri 2014, Bernini et al. 2006, Choong et al. 1992, Feller 1995, Lin et al. 2006, Lin and Lin 1985, Medina and Francisco 1997, Rao et al. 1994, Schmitt 2006, Tam et al. 1995, Tong et al. 2006, Christofoletti et al. 2013, Koch 2002, Wang et al. 2011, Lacerda et al. 1986,Medina et al. 2001, Nordhaus 2004, Nordhaus et al. 2011, Reise 2003, Woodroffe et al. 1988
leaf nitrogen (N) retranslocation prior to leaf senescence	Reise 2003
leaf oxalate content per leaf dry mass	Koch 2002
leaf phenolics content (polyphenol) per leaf dry mass	Christofoletti et al. 2013
leaf phosphorus (P) content per leaf dry mass	Ahmed et al. 2010, Bernini et al. 2006, Christofoletti et al. 2013, Feller 1995, Lin and Lin 1985, Medina and Francisco 1997, Tam et al. 1995, Medina et al. 2001, Reise 2003, Woodroffe et al. 1988
leaf phosphorus (P) retranslocation prior to leaf senescence	Reise 2003
leaf photosynthesis rate per leaf area	Chen et al. 2008, Clough and Sim 1989, Jiang et al. 2017, Li et al. 2016, Lugo et al. 2007, Mehlig 2001, Nandy (Datta) et al. 2005, Sobrado 2000
leaf potassium (K) content per leaf dry mass	Ahmed et al. 2010, Bernini et al. 2006, Christofoletti et al. 2013, Feller 1995, Lin and Lin 1985, Tam et al. 1995, Medina et al. 2001, Woodroffe et al. 1988
leaf sclerophyly index	Choong et al. 1992
leaf sodium (Na) content per leaf dry mass	Ahmed et al. 2010, Feller 1995, Lacerda et al. 1986, Tong et al. 2006, Wang et al. 2011, Medina et al. 2001, Woodroffe et al. 1988
leaf soluble tannins per leaf mass	Tong et al. 2006
leaf sulphur (S) content per leaf dry mass	Bernini et al. 2006, Christofoletti et al. 2013, Medina et al. 2001, Koch 2002
leaf thickness	Arrivabene et al. 2014, Choong et al. 1992, Das and Ghose 1996, Poompozhil and Kumarasamy 2014, Saenger and West 2016, Sheue et al. 2003, Yuanyue et al. 2009, Zimmer M unpubl. Data
leaf total aminoacid content per leaf dry mass	Koch 2002
leaf total carbohydrates per leaf dry mass	Lacerda et al. 1986, Tong et al. 2006
leaf total organic carbon per leaf dry mass	Schmitt 2006
leaf toughness	Choong et al. 1992, Zimmer M unpubl. data
leaf transpiration rate per leaf area	Mehlig 2001, Nandy (Datta) et al. 2005
leaf water content per leaf area	Ball 1988, Okello et al. 2014, Wang et al. 2011
leaf water content per leaf dry mass	Ball 1988, Chen and Ye 2008, Choong et al. 1992, Feller 1995, Lacerda et al. 1986, Saenger and West 2016, Tong et al. 2006
leaf zinc (Zn) content per leaf dry mass	Bernini et al. 2006, Feller 1995, Christofoletti et al. 2013
maximum salinity	Smith 1992
plant absolute maximum height	Chen and Twilley 1998, Duke and Jackes 1987, Duke et al. 2010, Ellison et al. 2010, Ellison et al. 2010, FAO Ecocrop 2017, Kathiresan et al. 2010, Khan et al. 2009, NParks 2017, Giesen et al. 2007
plant mean maximum height	Duke 2010, [Bibr B3666977][Bibr B3665525]
pneumatophore C/N ratio	Koch 2002
pneumatophore carbon content per unit dry mass	Koch 2002
root C/N ratio	Koch 2002
root carbon (C) content per unit dry mass	Koch 2002
root nitrogen (N) content per unit dry mass	Koch 2002
root porosity	Cheng et al. 2012, McKee 1996
root to shoot ratio	Reise 2003
seed air-dried mass	Royal Botanic Gardens Kew Seed Information Database (SID) 2017
seed C/N ratio	Nordhaus 2004
seed fresh mass	Royal Botanic Gardens Kew Seed Information Database (SID) 2017
seed litter carbon (C) content per unit dry mass	Nordhaus 2004
seed litter nitrogen (N) content per unit dry mass	Nordhaus 2004
wood density	Zanne et al. 2009
